# A review of food additives to control the proliferation and transmission of pathogenic microorganisms with emphasis on applications to raw meat-based diets for companion animals

**DOI:** 10.3389/fvets.2022.1049731

**Published:** 2022-11-10

**Authors:** Samuel S. Kiprotich, Charles G. Aldrich

**Affiliations:** Department of Grain Science and Industry, Kansas State University, Manhattan, KS, United States

**Keywords:** *Salmonella*, raw pet food, companion animals, raw meat-based diets (RMBDs), essential oils, organic acids

## Abstract

Raw meat-based diets (RMBDs) or sometimes described as biologically appropriate raw food (BARFs) are gaining in popularity amongst dog and cat owners. These pet guardians prefer their animals to eat minimally processed and more “natural” foods instead of highly heat-processed diets manufactured with synthetic preservatives. The market for RMBDs for dogs and cats is estimated at $33 million in the United States. This figure is likely underestimated because some pet owners feed their animals raw diets prepared at home. Despite their increasing demand, RMBDs have been plagued with numerous recalls because of contamination from foodborne pathogens like *Salmonella, E. coli*, or *Campylobacter*. Existing literature regarding mitigation strategies in RMBD's for dogs/cats are very limited. Thus, a comprehensive search for published research was conducted regarding technologies used in meat and poultry processing and raw materials tangential to this trade (e.g., meats and poultry). In this review paper, we explored multiple non-thermal processes and GRAS approved food additives that can be used as potential antimicrobials alone or in combinations to assert multiple stressors that impede microbial growth, ultimately leading to pathogen inactivation through hurdle technology. This review focuses on use of high-pressure pasteurization, organic acidulants, essential oils, and bacteriophages as possible approaches to commercially pasteurize RMBDs effectively at a relatively low cost. A summary of the different ways these technologies have been used in the past to control foodborne pathogens in meat and poultry related products and how they can be applied successfully to impede growth of enteric pathogens in commercially produced raw diets for companion animals is provided.

## Introduction

The domestication of the modern dog (*Canis lupus familiaris*) has resulted in a remarkable shift from the diet that its ancestor the wolf derived sustenance through scavenging and hunting small prey (1). The 2021–2022 American Pet Products Association (APPA) national pet owners survey reported that 70% of United States households owned a pet, which equates to 90.5 million homes (2). Because of anthropomorphism, there is an increasing number of pet owners who consider their animal a family member (3–6). Thus, the shift in human dietary choices due to increasing health consciousness are reflected in the ingredients pet owners prefer their animal consumed, thus there has been an increase in demand in foods that are considered “raw” and/or minimally processed (1, 7–9).

Raw meat-based diets (RMBDs) are a subset of minimally processed commercial diets (MPCD) or minimally processed home diets (MPHD) for companion animals and consist of raw or uncooked proteins sourced from animals such as lamb, pork, poultry, beef, venison, organ meats or offal, and supplemented with vegetables, tubers, dairy, or eggs (10, 11). However, the scope of this review will focus on RMBDs that are manufactured and marketed commercially in fresh and frozen forms. The market for MPCD diets is estimated at $120 million (11) of which RMBDs are a subcategory with estimates exceeding $33 million as of 2019. The feeding of companion animals with RMBDs is becoming increasingly popular because pet owners perceive these diets as “natural” and therefore presumed to provide additional health benefits to their animals, including but not limited to improved oral health, skin and coat compared to when these animals are fed conventionally heat sterilized foods manufactured through canning or extrusion (12–14). The shift toward RMBDs has resulted from concern that commercially available pet foods are over cooked when they are produced by extrusion (kibbles), baking (treats), or through canning (wet loaf and chunks and gravy styles). All undergo extremes in heat treatments during manufacturing to increase digestibility and assure microbial safety. However, these high temperatures are associated with increased degradation of some nutrients and formation of undesirable and potentially harmful compounds such as advanced glycation products (AGEs) (15–19). This tends to reinforce the argument for raw, and/or minimally processed pet food products (20–22). Presuming of course that safety can be assured by other means.

Typically, RMBDs are formulated with proteins from chicken, beef, lamb, duck, veal, and venison, organs like heart or liver, and are supplemented with bones, dairy products, fish, vegetables, fruits, and plant oils (10, 11). Vitamins and trace minerals may be added to these diets to adjust for any micronutrient shortcomings. Characteristically, these ingredients are ground and mixed into a batter and formed into patties, nuggets or placed into trays for commercial sale. Some pet owners prepare RMBDs from their homes because these diets are often expensive and not widely available in stores. Some pet owners opt to prepare these diets themselves because of the mistrust they have for “big” pet food companies due to numerous product recalls associated with aflatoxins and emerging research highlighting the ill health effects associated with animal consumption of AGEs, present in ultra-processed commercial diets (11). Regardless, the goal is that these diets meet the animal's nutrient requirements for amino acids, fatty acids, minerals, and vitamins.

Presuming nutrition can be met, the rest of the focus on these diets is safety and how to reduce microbial contamination by enteric foodborne pathogens such as non-typhoidal *Salmonella* spp. or *Campylobacter* that are inherently found in meat and poultry products. This is because raw diets cannot be heat processed, fermented, rendered, purified, extracted, or hydrolyzed by enzymolysis (23), thus leaving few avenues for efficient non-thermal antimicrobial interventions. Moreover, numerous studies have shown that RMBDs produced without adequate kill-steps are important vehicles for the transmission of pathogens to companion animals and to their human owners, during handling of food, or *via* cross-contamination with contact surfaces (24, 25). Foodborne enteric pathogens such as *Salmonella* spp., *Campylobacter jejuni, Listeria* spp., *Yersinia* spp., and *Escherichia coli* have been isolated from some commercial RMBDs globally (24, 25). Supplementary Table 1 provides a summary of pet food product recalls and withdrawals that were minimally processed due to contamination with foodborne pathogens reported by the Food and Drug Administration (FDA) from January 2017 to March 2021.

Raw meat-based diets, contaminated with foodborne pathogens have been linked to pathogenesis of certain diseases in pets for instance: Stiver et al. (25) prepared a case report of two cats that were diagnosed with *Salmonella* gastroenteritis and septicemia after necropsy, having been fed a home prepared RMBD. Morley et al. (24), observed cases of *Salmonella enterica* infections in a greyhound breeding facility that consumed raw diets and van Dijik et al. (26) reported that a dog fed with wild rabbit (hare) had tested positive for brucellosis. Although most healthy cats and dogs do not get ill from consuming contaminated RMBDs, some remain asymptomatic upon infection, and thus might shed the pathogen into the environment if animal excreta are not appropriately disposed (27, 28). Reports about the transmission of enteric foodborne pathogens from RMBDs to humans are still few with infections widely under-reported (24, 28). The CDC linked four outbreaks of multi-drug resistant *Salmonella* infections to raw turkey intended for feeding pets (29). Investigations by Public Health England (PHE) of the UK in (30) also linked an outbreak of Shiga toxin producing *Escherichia coli* (STEC) O157:H7 to contaminated raw pet food. Furthermore, antibiotic resistant strains of *Enterobacteriaceae* have been isolated from raw meats (beef, poultry, and fish) in retail shops by the World Health Organization (WHO). To corroborate WHO findings, Baede et al. (31); Jans et al. (32) reported that *E. coli* isolated from RMBDs exhibited similar resistance mechanisms as antimicrobial isolates that had been isolated from food production animals such as cattle, and pigs. However, it is worth noting that the transmission of enteric foodborne pathogens from RMBDs and companion animals fed these diets is a complex phenomenon to describe. This is because companion animals like dogs have a unique relationship with their environment and thus may nibble at objects, wild animal excreta or dead animal matter contaminated with any pathogen during normal daily activities not associated with the meal, thus complicating the process of tracking and analyzing the risk factors associated with RMBDs. Therefore, the purpose of this review is to investigate the different non-thermal methods of microbial control that have been successfully applied to meat and poultry while exploring alternative ways that these technologies can be employed to control and impede the proliferation of foodborne pathogens in RMBDs for companion animals.

## Contamination of raw meat-based diets

The choice of ingredients and the process of manufacturing RMBDs results into products that are highly perishable because they have a relatively high pH (5.5–6.5) and water activity of >0.98 (33). Animal and poultry carcasses are natural reservoirs of enteric foodborne pathogens such as *Salmonella* and *E. coli*, although muscle from healthy animals is sterile. These pathogens find their way into RMBDs because upstream harvesting techniques do not preclude fecal pathogens completely (34) and most processes do not involve an efficient pasteurization process and rely on the microbial quality of their ingredients and freezing/refrigeration of the product to control microbial growth during transportation or storage (10, 33). Contamination of RMBDs by foodborne pathogens such as *Salmonella, Campylobacter*, and enterohemorrhagic *E. coli* is not only a public health threat, but it leads to multiple product recalls annually which are also a significant financial loss to pet food manufacturers. The U.S. Department of Agriculture, Food Safety Inspection Service (USDA-FSIS) considers foodborne pathogens as adulterants in human foods whereas the FDA, the regulatory body for pet foods effectively have a zero-tolerance policy for enteric pathogens such as *Salmonella*, Shiga toxin-producing *E. coli* (STEC) and *Listeria monocytogenes* in commercial pet food, making the manufacturing and commercialization of RMBDs a herculean task. Discussed below are some of the most feasible non-thermal antimicrobial interventions that can be implemented in commercial pet food manufacturing plants to enhance the microbial safety of RMBDs.

## High pressure pasteurization (HPP)

There are several non-thermal pasteurization technologies currently available such as irradiation and ultrasonication that could theoretically be used to pasteurize RMBDs. The pet food industry in the United States relies heavily on high-pressure pasteurization (HPP) as the main technology for microbial inactivation in RMBDs (35). High pressure pasteurization utilizes hydrostatic force derived from the compression of water (or any incompressible fluid) applied to a food product intended for pasteurization (36, 37). The pressure used during HPP ranges between 100 and 1,000 MPa and system temperatures ranges between 4 and 90°C for a short duration (a few seconds or minutes) depending on the microbiological quality of the product being pasteurized (38). Unlike thermal pasteurization, HPP has several benefits in that the pressure is transmitted uniformly across the product, has a low environmental impact (low energy consumption and gaseous emissions), preserves heat labile nutrients like vitamins, pigments, antioxidants, and flavor/volatile compounds (39–41). The demand for clean label, minimally processed human/animal food products is on the rise, and HPP offers an alternative to using extensive heat processing or synthetic food additives to ensure safety and prolong shelf life of a product (38, 41).

High pressure pasteurization technology is a promising antimicrobial intervention strategy currently being employed as a microbial inactivation step to address microbiological hazards and ensure compliance with federal food safety regulations (39). Raw meat-based diets for companion animals utilize raw meat as their main source of protein. This meat does not undergo any pasteurization or cooking step to kill pathogenic or spoilage bacteria which makes HPP a prime candidate for RMBDs. The biological composition of raw meat (high moisture, fat, and protein) makes it highly perishable and an important vehicle for pathogen transmission, thus safety and quality concerns are a high priority (38, 40). When spoilage bacteria contaminate meat, they metabolize low molecular weight compounds like glucose, amino acids, and lactate to produce off-odors, sliminess, and discolorations associated with putrefaction. This putrefaction affects the organoleptic, visual, and nutritional quality of raw pet food. Beyond food safety, it is imperative that the proliferation of these spoilage microbes be controlled.

### Mechanism of microbial inactivation using HPP

High pressure pasteurization relies on the principles of Pascal's law which states that compression applied on one part of a liquid medium can be transmitted instantaneously through all parts of the mass being treated (37). The application of high pressure might lead to a slight increase in temperature, and thus the net effect of HPP might be a combination of heat, pH change, or other microbial stressors that could achieve cellular disruption and inactivation. Either way, it is an example of hurdle technology that involves increasing the number of barriers for microorganism growth and survival. In various applications, HPP has been used successfully to inactivate enzymes, and pathogenic and spoilage microorganisms (36, 38, 39). The effects observed on meats treated with high pressure are dependent on the amount of pressure applied, temperature, and the duration (time) of the process. Thus, components of meat sensitive to high pressures such as myosin and myoglobin may limit the application of HPP to fresh meats in favor of fermented, precooked, or restructured meats (38, 39) due to weeping and syneresis.

The mechanism in which high pressure processing kills or mitigates the growth of pathogenic and spoilage bacteria is *via* cellular injury. This leads to death or impedes the ability of the microbes to repair, resuscitate or grow. The events that lead to cell death by high pressure processing are not well-understood even though several bacterial species have been studied (39). High pressure processing carried out at ambient conditions and hydrostatic pressure held between 300 and 800 MPa showed significant inactivation of vegetative cells. The inactivation of vegetative cells was because of denaturation and unfolding of critical metabolic and physiological enzymes in the cytoplasm, happening simultaneously with cell membrane rapture resulting from phase transitions of the cytoplasmic fluids (36, 38, 39, 41). The method of inactivation is reliant on hydrostatic pressure and intrinsic and extrinsic factors associated with a given microorganism. For instance, synergism has been observed with increased pressure and increased adiabatic temperatures that potentiate the lethality process (39, 41, 42).

### Application of HPP in processing of RMBDs

There is limited published research investigating the use of HPP to inactivate enteric foodborne pathogens in RMBDs. Thus, to understand applicable research, we conducted a systematic search of the literature. The search was conducted by selecting key words, which were input into selected databases, and then the inclusion/exclusion criterion was established. The key words included “pet food,” “dog,” “RMBD,” “raw meat-based diet,” “raw pet food,” “BARF,” “meat,” “poultry,” “high pressure pasteurization,” “high pressure processing,” “HPP,” “ground meat,” “ground poultry,” “minced,” and “filets.” These key words were applied to Google Scholar and Scopus with no limit to years or language. Original research and review articles investigating the use of HPP in microbial inactivation of RMBDs and comminuted meats were considered in this section. Comminuted meats have an increased surface area for pathogen attachment and proliferation, which decreases the antimicrobial efficacy of HPP treatments. Articles in book chapters, patents, trade publications, extension bulletins, and conference abstracts were excluded from this section.

Pasteurization of meat and poultry using HPP has been demonstrated as an effective process to control spoilage and pathogenic bacteria in meat and poultry products (38, 43–48). These studies demonstrated that whole chunks/cuts of meat were easier to pasteurize as the interior was sterile compared to when comminuted meats were used. Serra-Castelló et al. (49) reported that the antimicrobial efficacy of HPP (450–750 Mpa) against *Salmonella* inoculated in RMBDs formulated with lactic acid (0–7.2 g/kg) was enhanced as they observed log reductions ranging from 0.76 to 9.0 Log CFU/g depending on different combination of factors (time, pressure, and lactic acid concentrations). However, Simonin et al. (50) conceded that high-pressure treatments above 400 MPa resulted in significant reduction in microbial counts but induced adverse changes in the quality attributes of meat such as color, texture, and accelerated lipid oxidation.

The process of comminuting meat and poultry products increases surface area for microbial attachment and facilitates the redistribution of spoilage and pathogenic bacteria making pasteurization by HPP less effective. For instance, Sheen et al. (51) was able to achieve a 5 Log CFU/g reduction after treating 90 g of ground chicken using HPP at 500 Mpa for 10 min. The log reduction achieved by Sheen et al. (51) was notable but could not be feasibly applied industrially to pasteurize ground chicken. This is because high levels of pressure are required to inactivate pathogens, increasing energy costs which are exacerbated by the low throughput (90 g/10 min) that was reported in this study.

New studies indicate that the antimicrobial efficacy of HPP can be potentiated through combinations with food additives such as organic acids and essential oils to achieve higher log reductions while keeping the required pressure relatively low whilst increasing the shelf-life and safety of the meat (52, 53). Combination of HPP and organic acidulants or essential oils allows for the destruction of sub-lethally injured bacterial cells that often resuscitate and multiply, leading to product recalls. However, HPP operations may require that products be transported in chubs into a “clean room” for reformation, which might result in recontamination of the product during handling, packaging, or transit. Thus, the costs and contamination risks associated with HPP can be avoided through the utilization of generally recognized as safe (GRAS) food additives since they are relatively inexpensive, can be uniformly distributed in a product and have a residual antimicrobial effect which enhances safety of the RMBD products over prolonged periods of time compared to HPP.

## Use of generally recognized as safe (GRAS) food additives to control foodborne pathogens in raw meat-based diets

There is limited research regarding organic acidulants to control foodborne pathogens in RMBDs. To understand the published work that might be applicable, we conducted a two-part systematic search of the literature. The search was organized by selecting key words, identifying the appropriate databases, and determining inclusion and/or exclusion criterion. Search one key words included “pet food,” “dog,” “RMBD,” “raw meat-based diet,” “raw pet food,” and “BARF,” applied to Google Scholar and Scopus with no limit to years or language. Original research, and review papers with synthesis of new findings were included, and book chapters, patents, trade publications, extension bulletins, and conference abstracts were excluded. Search two key words included “essential oils,” “organic acids,” “bacteriophages,” “ground,” “minced,” “cubed,” “trimmings,” “skin,” “filets,” “beef,” “chicken,” “lamb,” “pork,” and “turkey” was also applied to Google Scholar and Scopus with no limitations to years and language. Only research and review articles evaluating the antimicrobial efficacy of food additives in comminuted meats were considered for this section of the review paper. Cases where whole chunks and comminuted meats were analyzed concurrently were also considered and included in the summary tables. This is because comminuted meats have increased surface area for pathogen attachment, proliferation, and difficulty of decontamination as these mimicked the way RMBDs are manufactured and retailed. Articles in book chapters, patents, trade publications, extension bulletins, and conference abstracts were also excluded from the summary tables.

## Use of organic acidulants to control enteric pathogens in RMBDs

Organic acidulants are considered by the Code of Federal Regulations (CFR) as generally recognized as safe (GRAS) additives. They have been commonly applied to animal and poultry meats because these acids are relatively inexpensive and have been demonstrated to be efficient antimicrobials (54, 55). Examples of these acids are lactic, citric, succinic, propionic, malic, and acetic and their salts. Most GRAS organic acids do not have a daily (maximum) acceptable intake for humans or animals which increases their applicability. However, their dosage is limited by their negative impact on organoleptic and color attributes of meat and poultry products. Most organic acids are described as weak acids because they do not fully dissociate in water but rather dissociate in a pH-dependent manner (56). When organic acids are added to meats, the pH of the meat is lowered to that which is equal to or lower than the acid dissociation constant (pKa) of the acid, resulting in an increased concentration of protonated acid which is responsible for the antimicrobial activity of the organic acid (56).

There are two primary mechanisms by which organic acids elicit antimicrobial activity: first, by cytoplasmic acidification which impedes ATP production and regulation, and secondly through accumulation of dissociated anions from the organic acid to toxic levels affecting cell physiology and metabolism (56). A transmembrane gradient may be created if the cytoplasmic pH is higher than that of the surrounding membrane leading to diffusion of undissociated acid through the cell membrane. The more alkaline pH of the cytoplasm then encourages the dissociation of the acid yielding anions and protons (57, 58). Accumulation of undissociated acid in the cytoplasm was associated with shifting the cytoplasmic pH which affected enzymatic activity, protein, and nucleic acid synthesis (59). Lactic acid was reported to make the cell membrane more permeable in Gram-negative bacteria, causing a leakage of lipopolysaccharides (60). Alakomi et al. (61), further reported that the chelating properties of citric and malic acids caused an intercalation of the outer membrane of *Salmonella*. Additionally, mold inhibitors such as sorbic acids contain more hydrophobic compounds and have been reported to increase the permeability of the membranes while interfering with membrane proteins; thus, helping to inhibit mold (62). However, recent research shows that the mechanisms of cellular inhibition or death by organic acidulants are not unilateral as these acids interact with different bacterial membranes and structures creating crippling hurdles that lead to either growth inhibition or inactivation. This would suggest that one mechanism is inadequate to accurately describe the mode of action for a singular organic acid as a food additive for control of spoilage and (or) enteric pathogens in human and animal foods (63).

### Potential application of organic acids in RMBDs

Generally recognized as safe (GRAS) organic acids such as acetic (21CFR184.1005), citric (21CFR184.1033), and lactic (21CFR184.1061) acids have been approved by the FDA for direct addition to manufactured foods as antimicrobial interventions on meat carcasses and derived cuts pre- and post-chilling at concentrations of <5% (54, 64). Studies regarding the antimicrobial efficacies of these organic acids in the meat industry have been widely conducted and reported. Lactic acid at 150 mM was vacuum infused into boneless/skinless chicken breast cubes that had been inoculated with 10^8^ Log CFU/g of *S*. Typhimurium and stored at 4°C and a 2.5 log reduction was observed by the 6th day while there were no significant reductions on day 9 and 12 (65). Over et al. (65) further tested different organic acids, citric, malic, and tartaric acids at the same concentration of 150 mM using the same procedure described above. By day 6, the initial inoculum of *S*. Typhimurium had dropped from its initial concentration of 10^8^ Log CFU/g to just 10^2^ Log CFU/g and were undetectable by day 9. Citric acid was just as effective as acetic acid in the control of *S*. Typhimurium compared to lactic acid, but its application was limited by the negative impact on the quality attributes of the chicken.

Most studies have evaluated the antimicrobial efficacy of organic acids in ridding surfaces of pathogenic contaminants in both animal and poultry meats because the inside tissues of the meat are considered sterile. However, when these tissues/cuts/trimmings are ground, a new challenge arises when utilizing organic acids as antimicrobial interventions due to an increase in surface area available for microbial attachment and proliferation. For example, Harris et al. (66) inoculated beef trimmings with strains of *Salmonella* or *E. coli* O157:H7 at a concentration of 4.0 Log CFU/g and then ground the beef trimmings with two different levels of lactic acid and citric acid at 2.0 and 4.0%, respectively. Microbial analysis of the ground inoculated meats revealed a 2.5 log reduction in *E. coli* O157:H7 and a 1.5 log reduction of *Salmonella* after the ground meats were held frozen for a month.

Published studies (Table 1) that utilized organic acids at various doses to control enteric foodborne pathogens in meat/poultry products and the log reductions that occurred in the microbial challenge studies. Overall, the log reductions reported in Table 1 ranged from 0.3 to 4.3 Log CFU/g. When whole chunks/cuts of meat were treated with organic acids, and challenged against a foodborne pathogen, relatively higher log reductions were observed than when ground meats and sausages were used in a study. Also, the types of acidulant used to treat the meat or poultry may affect the log reductions observed, for instance, when Hamby et al. (67) treated beef cubes with both acetic and lactic acid at 1.0%, the latter resulted in a significant reduction of the aerobic plate counts (APC). However, Tamblyn and Conner (73) reported no differences in log reductions when they treated chicken breast skins with malic or tartaric acids at 1.0%. The log reductions were also dependent on the dose of acidulants used as higher concentrations of acid resulted in higher log reductions.

**Table 1:** Summary of antimicrobial efficacy for organic acids at various doses used to control spoilage and pathogenic bacteria in meat and poultry products.

**Food/meat**	**Organic acid**	**Dose**	**Microorganism**	**Log reduction**	**References**
Ground Beef	Acetic acid	2.0%	S. Typhimurium	1.5 Log CFU/g	(66)
	Lactic acid	4.0%	*E. coli* O157:H7	2.5 Log CFU/g	
Beef cubes	Acetic acid	1.0%	Aerobic Plate counts	1.8 Log CFU/cm^2^	(67)
	Lactic acid	1.0%		4.3 Log CFU/ cm^2^	
Broiler chicken skins	Acetic acid	4.0%	*Salmonella*	2.0 Log CFU/ cm^2^	(68)
Raw Chicken	Sodium acetate	6 g/chicken	Enterobacteriaceae	3.0 Log CFU/chicken	(69)
Fresh pork sausage	Sodium citrate	1.5%	S. Kentucky	0.3 Log CFU/g	(70)
Beef tissue	Lactic acid	2.0%	S. Typhimurium	1.2 Log CFU/g	(71)
Skinless chicken breast	Lactic acid	150 mM	S. Typhimurium	2.5 Log CFU/g	(65)
Fresh pork	Lactic acid	3.0%	S. Typhimurium	2.33 Log CFU/cm^2^	(72)
Chicken breast skin	Malic acid	1.0%	S. Typhimurium	2.16 Log CFU/cm^2^	(73)
Chicken breast skin	Tartaric acid	1.0%	S. Typhimurium	2.16 Log CFU/cm^2^	(73)

The broad potential applicability of organic acids in food products to enhance safety and quality is complicated because the high acid and low pH usually alters the sensory properties of meats and poultry. Application of acids directly at higher concentrations alters the quality of meat products resulting in changes in meat color and syneresis perceived as negative by pet owners (82). Consequently, there needs to be a means to slowly deliver the acidulants into the meat product to ensure minimal changes in product quality. For instance, encapsulating organic acidulants with soluble and edible vegetable oil films allows for a “slow release” mechanism, melting and releasing the acid into the meat at a slow and controlled rate, avoiding the acid shock effect observed when direct/raw acids are applied to meat (82). Ultimately, one way to increase the utilization of organic acids in RMBDs as antimicrobial interventions would be through encapsulation.

## Use of essential oils as antimicrobials in RMBDs

**Figure 1:**
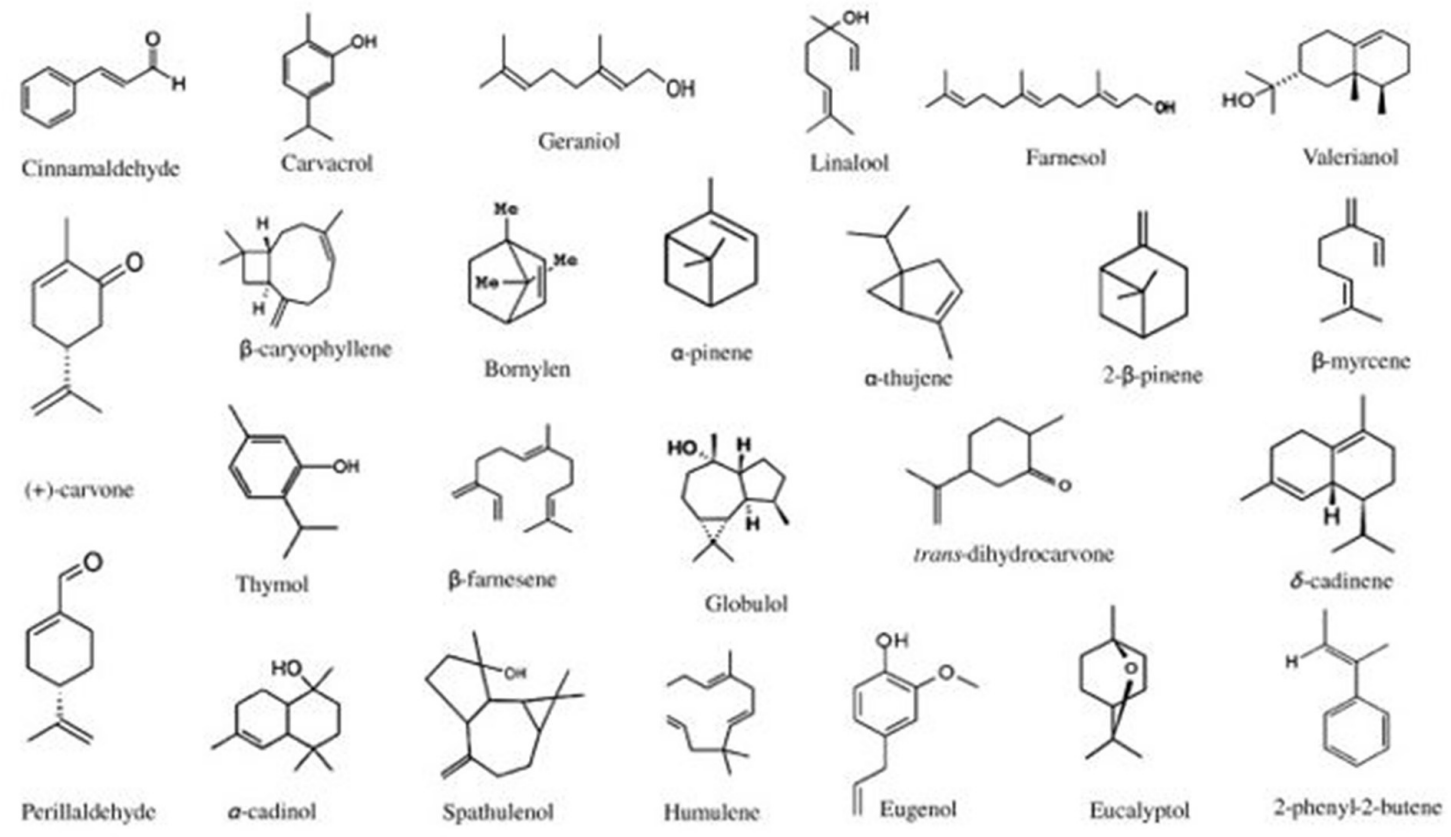
Chemical structures of components of essential oils [adopted from Bajpai et al. (88)].

Essential oils (EOs) are types of phytochemicals produced by aromatic plants primarily for defense against microbial invasion (83–85). These EOs consist of many components, such as terpenes, alcohols, acids, esters, aldehydes, and ketones (83, 86). Of these components, the volatile bioactive components are responsible for the antimicrobial activity of EOs (87). Examples of EOs are thyme, rosemary, cinnamon, eucalyptus oils, etc. Furthermore, certain components of these oils have been extracted and used as antimicrobials such as thymol, eugenol or cinnamaldehyde. As an example, the adaptation of the list of EOs by Bajpai et al. (88) (Figure 1) shows the different chemical structures of components that make up EOs.

To achieve microbial decontamination by these EOs, a few theories on their mechanism of action have been proposed. Many studies have demonstrated that components of EOs work synergistically to control the proliferation of microorganisms. Burt (89) reported that the hydrophobicity of the components of EOs increased cell permeability which allowed antimicrobial compounds to enter the cell cytoplasm. Essential oils contain different forms of phenols that disrupt cell membranes increasing permeability, leakage of cell contents, inhibition of ATPases which affects ATP production, and ultimately leading to cell death (88, 90, 91).

### Application of essential oils in raw meat-based diets

Biochemical reactions such as lipid oxidation, autolytic enzymatic spoilage, and microbial spoilage result in significant losses of meat and poultry products along the production chain and have substantial economic and environmental impacts (92, 93). Essential oils (EOs) and their components can be used as a natural alternative to synthetic preservatives and there are several studies that have explored their use in meat and poultry products (76, 84, 88). Spoilage microorganisms that lead to deterioration of meat quality include, *Pseudomonas, Acinetobacter, Lactobacillus spp., Enterobacter* etc., yeast, and mold (83). These microorganisms' metabolic activity results in the formation of off-flavors, odors, and changes in color which are associated with deterioration in meat products. In addition to spoilage organisms, meat potentially harbor pathogenic enteric microbes such as *Salmonella spp., Staphylococcus aureus, Listeria monocytogenes, Clostridium perfringens, Clostridium botulinum*, Enterohemorrhagic *E. coli*, and *Campylobacter spp*. that are inherent in meat and can be controlled using EOs (91).

Table 2 shows a summary of published studies in which different meat and poultry products were treated with EOs and challenged against enteric foodborne pathogens. The data in Table 2 show the type and dose of EO, the kind of pathogen or serovar challenged against the EO, and the log reduction observed after microbial analysis. The log reductions observed varied from 1 to 3 Log CFU/g when the EOs were challenged against different pathogens or their serovars. However, the difference in antimicrobial efficacies of the EOs observed in the various studies could be attributed to interaction of various factors such as type of meat, dose of EO, the strain/serovar of a pathogen or the duration of pathogen exposure to the EOs. The interaction of some of these factors resulted in varying log reductions across studies and among similar pathogens. For instance, Kiprotich et al. (76) reported a 3.48 Log CFU/mL reduction when *Salmonella enterica* was challenged against 0.5% (v/v) thyme oil. However, Boskovic et al. (79) applied 0.3% thyme oil and only observed a 1.69 Log CFU/g reduction. The difference between these two studies was that Kiprotich et al. (76) added thyme oil into lemon juice and supplemented the mixture with *Yucca schidigera*, a natural emulsifier, and allowed the mixture to stand at 23°C for 8 h when microbial analysis was performed; whereas Boskovic et al. (79) pulled a vacuum on the packaging of the minced meat and stored their samples at 3 ± 1°C for 15 days. The difference in the results obtained in these two studies can be attributed to the synergistic effects of the different conditions offered to the essential oils. The other avenue would be to combine different types of EOs because each may contain different concentrations and modes of action. For instance, Thannisery and Smith (77) combined thyme oil and orange essential oil at 0.5% (v/v) each and achieved a 2.6 Log CFU/mL of *Salmonella* Enteritidis and a 3.6 Log CFU/mL reduction of *Campylobacter coli* in chicken breast meat. Combinations of EOs and other antimicrobial strategies such as emulsifiers, modified atmospheric packaging or refrigeration might increase the applicability of EOs by fostering a synergistic, complimentary antimicrobial effect, which in turn circumvents the strong flavors and damage to sensory properties of food usually associated with application of higher concentrations of EOs (76, 94, 95).

**Table 2:** Summary of antimicrobial efficacy for essential oils at various doses used to control spoilage and pathogenic bacteria in meat and poultry products.

**Food/meat**	**Essential oil**	**Dose of EO**	**Microorganism**	**Log reduction**	**References**
Beef filets	Oregano oil	0.8%	*L. monocytogenes*	2 Log CFU/g	(74)
			Lactic acid bacteria	3 Log CFU/g	
Minced meat	Oregano oil	1.0%	Total viable counts	1 Log CFU/g	(75)
Chicken breast cubes	Thyme oil + lemon juice	0.5%	*S. enterica*	3.48 Log CFU/cube	(76)
Broiler breast meat	Thyme oil + orange essential oil	0.5% each EO	*S*. Enteritidis	2.6 Log CFU/mL	(77)
			*Campylobacter coli*	2.6 Log CFU/mL	
Minced sheep meat	Oregano oil	0.9%	*S*. Enteritidis	2.53 Log CFU/g	(78)
Minced pork meat	Thyme oil + vacuum conditions	0.3%	*S. enterica* (Infantis, Typhimurium, Montevideo, Enteritidis)	1.69 Log CFU/g	(79)
		0.6%		2.00 Log CFU/g	
		0.9%	Cocktail	3.85 Log CFU/g	
Ground chicken	Carvacrol	0.1%	*S. enterica* (Heidelberg, Typhimurium, Montevideo, Kentucky)	0.12 Log CFU/g	(80)
	Mustard oil	0.75%	Cocktail	0.31 Log CFU/g	
Minced meat	Thyme oil	0.1%	Mesophilic bacteria (*Salmonella, E. coli, L. monocytogenes*)	Log CFU/g	(81)
	Ginger oil	0.1%		1.3 Log CFU/g	

To apply EOs in RMBDs, they might have to be added to the product during the grinding and mixing process. A formula for most commercially available pet foods consists of ground meats with bones, tubers, vegetables, and fruits. In this form, they present a challenge to decontamination since surface treatment alone is not sufficient. Unlike whole chunks of meat or poultry which have been successfully decontaminated with EOs, grinding reduces particle size while increasing surface area for pathogen attachment and distribution throughout the product. Supplementary measures such as modified atmospheric packaging (MAP), freezing or vacuumizing might synergize the antimicrobial processes discussed above.

## Bacteriophages

Bacteriophages refer to host-specific viruses that parasitize bacteria by lysing, breaking, and penetrating through the cell membrane and multiplying inside the cell, causing its death (96, 97). Bacteriophages are ubiquitous in the environment, and highly specific making them ideal for the biocontrol of bacteria as they attack a wide range of spoilage and pathogenic microorganisms while maintaining their specificity (96, 98). These phages may belong to the Order Caudovirales, with their respective families including *Myoviridae, Siphoviridae*, and *Podoviridae* (99–101). Bacteriophages are increasingly being applied to liquid foods as an alternative to chlorine-based decontaminants which are associated with rising incidences of antimicrobial resistance (102).

The mechanism by which bacteriophages parasitize bacteria is based upon the specificity of the phage virus to a singular bacterial species or one very similar (98, 99, 103). Despite their ubiquity in the environment, a relatively small proportion of phage viruses possess the specificity required to bind with a target pathogen, thus their overall impact on the microbial ecosystem remains insignificant regarding negative effects (104, 105). As an example of bacteriophage specificity, Ricci and Piddock (106) demonstrated that ST27, ST29, and ST35 phages only bound to TolC receptors present on outer membranes of *Salmonella* serovars but were totally inactive against receptors found in the *Enterobacteriaceae* family. Whereas, some phages express a phenomenon described as “local adaptation,” that allows them to infect bacteria across several genera (105, 107, 108).

The phage attaches to specific receptors on the outer cell membrane and then injects itself by adsorption. Once in the cell, the phage will either follow a lytic or lysogenic lifecycle. The lytic or virulent cycle causes rapid cell death as the phage uses the cell to replicate (96). Daughter phages are released upon cell lysis to infect the next line of bacterium. For lysogenic phages they transfer their genome to bacterial cells and use the host replication which results in the transmission of phage genome through host daughter cells but does not result in cell death (101). Lytic phages minimize transduction of their genome into their host leading to cells resisting phage viruses (phage resistance) whereas lysogenic phages contribute to phage resistance as they transfer their genome through the host cells (99, 101). From the mechanisms of action discussed above, lytic phages would be appropriate for use in therapeutic and antimicrobial interventions in both animal and human food.

### Application of bacteriophages to control pathogens in raw diets

The relationship between bacteria and phages is expressed as ratio, described as “multiplicity of infection (MOI),” and multiplicity of adsorption (MOA) which is a ratio of the phage forming units to colony forming units (PFU/CFU) (96, 98). This ratio allows for phages to be applied as an antimicrobial intervention with the efficacies of different phage concentrations determined by the number (CFU) of bacterial cells inactivated by a specific concentration of phage viruses (PFU) (105, 109–111). However, the concentration of bacterial cells has been shown to have no effect on the antimicrobial potency of the phages as demonstrated by Bigwood et al. (112) who increased the concentrations of *Salmonella* while keeping constant *Salmonella* phages (P7) and observed no difference in inactivation efficiency. Likewise, Bigwood et al. (112) increased the phage concentration from 1.8 × 10^4^ to over 5 × 10^8^ PFU/mL and observed increased inactivation of *Salmonella*, and vice versa when the phage concentration was lowered. Bacteriophages have been mainly applied to liquid foodstuffs, but progress has been made for application to solid foods. The current challenges of phage application are the development of resistance to phages by bacteria which necessitates the use of phage cocktails to control mutating (adapting) cells. Secondarily not all phages are recognized by the FDA as GRAS.

Phages are ubiquitous which allows for flexibility when they come into contact against a serotype of a spoilage or pathogenic bacterium. They offer an alternative non-thermal method to treat minimally processed or raw foods or ingredients. Studies that employed bacteriophages in meat and poultry to control enteric pathogens is summarized in Table 3. There was a higher log reduction of the pathogens challenged against the phages in whole chunks of meat compared to ground meat. The phages' antimicrobial activity also appeared to depend on the type of serovar of pathogen they were exposed to. For instance, Spricigo et al. (113) challenged *Salmonella enterica* serovar Typhimurium and Enteritidis inoculated in poultry meat against a phage solution at 10^9^ PFU/mL and observed a significant difference in log reduction (2.2 and 0.9 Log CFU/g, respectively). Also, when different types of phages were challenged against the same *Salmonella* serovar different log reductions were observed after treatment. Furthermore, Hungaro et al. (102) isolated bacteriophages from poultry feces and used them against *Salmonella* Enteritidis on chicken skin and reported a 1.0 Log CFU/cm^2^ reduction as an alternative to chlorine, a chemical disinfectant. Higgins et al. (114) sprayed carcasses of broilers and turkey inoculated with *Salmonella* Enteritidis with rinse water containing 10^9^ PFU/mL of PHL4 bacteriophages and reported a 93.0% (on broilers) and 58% (on turkeys) reduction of the initial concentration of pathogens compared to the control carcasses that were sprayed with only water.

**Table 3:** Summary of antimicrobial efficacy for bacteriophages used to control enteric foodborne pathogens in meat and poultry products.

**Food/meat**	**Bacteriophage**	**Phage dose**	**Target pathogen**	**Log reduction**	**References**
Ground beef	S16 and FO1a	10^9^ PFU/mL	S. Typhimurium S. Infantis S. Heidelberg	1.0 Log CFU/mL	(55)
Pig skin Poultry Fresh eggs	UAB_Phi20 UAB_Phi78 UAB_Phi87	10^10^ PFU/mL 10^9^ PFU/mL 10^10^ PFU/mL	S. Typhimurium S. Enteritidis S. Typhimurium S. Enteritidis S. Typhimurium S. Enteritidis	2.9 Log CFU/cm^2^ 2.2 Log CFU/cm^2^ 2.2 Log CFU/g 0.9 Log CFU/g 0.9 Log CFU/g 0.9 Log CFU/g	(113)
Poultry skin	*Podoviridae* (phiSE)	10^9^PFU/mL	S. Enteritidis	2.2 Log CFU/cm^2^	(102)
Broiler carcass Turkey	PHL4 PHL4	10^9^PFU/mL 10^9^ PFU/mL	S. Enteritidis S. Enteritidis	93.0% reduction 58.0% reduction	(114)

The application of bacteriophages is still limited by factors such as pH and temperature which affect their antimicrobial potency. For instance, Leverentz et al. (115) applied a specific phage cocktail to honeydew melon (pH 5.8) and apple slices (pH 4.2), stored at 5, 10, and 20°C. A 2.5–3.5 log reduction of *Salmonella* Enteritidis was observed on the honeydew slices that were stored at 5 and 10°C, whereas no significant log reduction was observed at 20°C. There was no significant reduction of *Salmonella* Enteritidis on the apple slices at any temperature level leaving the authors to hypothesize that the phages had been deactivated by low pH of the apple slices. The implication of their observation is that more acid-resistant phages need to be developed for application in low pH food systems or matrices if they are to be deployed as antimicrobial interventions.

## Summary

The high-pressure pasteurization and food additives discussed as interventions in this review have exhibited antimicrobial efficacies of varying successes against spoilage and pathogenic bacteria in poultry and meat products. However, commercialization and adoption of these novel interventions by the animal and pet food industry has been slow because of the varying antimicrobial efficacies obtained from using these technologies when applied to control enteric foodborne pathogens in meat and poultry products. Variation in experimental design, microbial strains, equipment, and outcomes have made the adoption and scale-up of these interventions difficult due to inadequate reproducibility of the results from these studies. For instance, different studies that utilized the same intervention (i.e., essential oil, organic acidulant, or bacteriophage) against a similar pathogen resulted in different results under comparable conditions (Tables 1–3). The lack of consistency makes standardization of these antimicrobial interventions difficult given that they are mainly applicable to minimally processed foods which are at a higher risk of being contaminated. Furthermore, effective pasteurization requires that higher doses of these non-thermal interventions be applied which can have undesirable effects on the sensory and nutritional attributes of a given pet food, warranting additional research to address palatability concerns.

A path forward is rooted in hurdle technology on the premise that combining technologies will act synergistically. Harnessing this synergism could allow for lower doses to be applied to products, may lower the negative impact on quality and sensory attributes of the treated foods and has the potential to increase consistency in effective pathogen control. Combinations of essential oils, high-pressure processing, and low pH tolerant phages should be developed which would allow the combination of organic acids and bacteriophages to become a reality. Improving the safety of RMBDs for companion animals, given the biological hazards discussed in this review will require a holistic approach. First, utilization of food additives like organic acids or essential oils considered as GRAS and “natural” should be a first step. Secondly, these interventions should be evaluated in combination by taking advantage of their different mechanisms of antimicrobial action.

Also, strategies like modified or controlled atmospheric packaging should be researched in addition to these new emerging technologies because air composition affects microbial life and, thus, it might introduce a stressor, impeding the growth of pathogenic microbes. Kinetic mechanistic maps of bacteria from different genera can help scale up these proposed antimicrobial interventions by highlighting the more robust and resistant microbes in the matrices of a RMBD. In conclusion, as the demand for RMBDs increases, so will safety challenges associated with them. Innovative and holistic approaches will need to be developed and utilized to address microbial safety and hazards associated with commercial RMBDs. Therefore, the antimicrobial interventions discussed in this review may be a framework for future research aimed at controlling foodborne pathogens in commercially manufactured RMBDs for companion animals.

## Author contributions

SK and CA came up with the idea for this review. SK wrote the manuscript. CA edited the final drafts of this review paper. All authors contributed to the article and approved the submitted version.

## Conflict of interest

The authors declare that the research was conducted in the absence of any commercial or financial relationships that could be construed as a potential conflict of interest.

## Publisher's note

All claims expressed in this article are solely those of the authors and do not necessarily represent those of their affiliated organizations, or those of the publisher, the editors and the reviewers. Any product that may be evaluated in this article, or claim that may be made by its manufacturer, is not guaranteed or endorsed by the publisher.
