# Supplementary material for: A review of food additives to control the proliferation and transmission of pathogenic microorganisms with emphasis on applications to raw meat-based diets for companion animals
Source: Front Vet Sci. 2022 Nov 10;9:1049731. doi: 10.3389/fvets.2022.1049731 (PMC9686358; doi:10.3389/fvets.2022.1049731)
Supplement: Supplementary file 1 [file Table_1.docx]

**Supplementary Table 1****. Recalls and withdraws of minimally processed pet foods and treats due to contamination with enteric foodborne pathogens reported by the Food and Drug Administration (FDA) between January 2017 and March 2021**

| Date | Brand-Names | Product-Description | Recall-Reason-Description | Company-Name | Terminated-Recall |
| --- | --- | --- | --- | --- | --- |
| 03/19/2020 | G & C Raw, Kim’s Special, Pat’s Cat | Raw dog and cat food | *Listeria* | G & C Raw, LLC | Terminated |
| 03/23/2020 | G & C Raw, more | Ground Lamb Dog Food and Ground Turkey Cat Food | Potential to be contaminated with *Listeria monocytogenes* | G & C Raw LLC | Terminated |
| 02/09/2018 | Raws for Paws | Ground turkey pet food | Potential to be contaminated with *Salmonella* | Raws for Paws | Terminated |
| 04/06/2020 | OC Raw Dog | Chicken, Fish, & Produce Dog Food | *Listeria* | OC Raw Dog, LLC | Terminated |
| 04/06/2020 | OC Raw Dog | Freeze Dried Sardines | Incorrect compliance guidelines | OC Raw Dog, LLC | Terminated |
| 11/13/2020 | Albright’s | Raw Dog Food Chicken Recipe for Dogs | Potential to be contaminated with *Salmonella* | Albright’s Raw Dog Food |  |
| 11/14/2019 | Quest | Beef Cat Food | May be contaminated with *Salmonella* | Go Raw, LLC |  |
| 03/06/2018 | Tucker's | 5lb Pork-Bison Box | Potential to be contaminated with *Salmonella* | Raw Basics, LLC | Terminated |
| 02/06/2018 | Companion, Dantley’s and more | Rawhide chew products | Possible chemical contamination | United Pet Group, a division of Spectrum Brands, Inc. | Terminated |
| 02/08/2018 | Various brands: American Beef hide, Digest-eeze, Healthy Hide, Healthy Hide Good n Fun, Healthy Hide Good n Fit | Rawhide dog chews | Potential Contamination | United Pet Group | Terminated |
| 03/20/2020 | Rad Cat | Rad Cat Raw Diet | Potential to be contaminated with *Listeria monocytogenes* | Radagast Pet Food, Inc. | Terminated |
| 04/07/2020 | Natural Selections and ZooLogics | Fresh raw meals for dogs | Due To Contamination *Salmonella* and Shiga Toxin-Producing *Escherichia coli* O128 | Darwin’s Natural Pet Products | Terminated |
| 04/05/2019 | Thogersen Family Farm | raw frozen ground pet food | Potential to be contaminated with *Listeria monocytogenes* | Thogersen Family Farm |  |
| 08/15/2019 | Texas Tripe | Raw frozen pet food | Potential to be contaminated with *Salmonella* and *Listeria monocytogenes* | Texas Tripe Inc. |  |
| 03/19/2020 | Performance Dog | frozen raw pet food | Potential to be contaminated with *Salmonella*. | Bravo Packing, Inc. | Terminated |
| 03/26/2018 | Blue Ridge Beef | Complete raw pet food | Potential of contamination with *Salmonella* and *Listeria monocytogenes* | Blue Ridge Beef |  |
| 03/01/2018 | Blue Ridge Beef | Raw pet food for cats | Due to Possible *Salmonella* and L. Mono Contamination | Name Blue Ridge Beef |  |
| 04/08/2020 | Rad Cat | Free-Range Chicken and Turkey Recipes (Raw Diet) for Cats | Potential to be contaminated with *Listeria monocytogenes* | Radagast Pet Food, Inc. | Terminated |
| 03/24/2020 | Rad Cat | Raw Diet Cat Food (Free Range Chicken; Pasture-Raised Venison) | Potential to be contaminated with *Listeria monocytogenes* | Radagast Pet Food, Inc. | Terminated |
| 03/02/2018 | Steve’s Real Foods | Raw frozen dog food turkey canine recipe | *Salmonella* | Steve’s Real Foods | Terminated |
| 03/03/2021 | Bravo Packing, Inc. | Ground Beef and Performance Dog, frozen raw pet food | Potential for *Salmonella* and *Listeria monocytogenes* | Bravo Packing, Inc. |  |
| 01/17/2017 | Blue Ridge Beef | Raw Dog and Cat food | potential to be contaminated with *Listeria monocytogenes* | Blue Ridge Beef | Terminated |
| 04/06/2020 | TRUDOG | Pet food-freezed dried raw beef topper | *Salmonella* | TruPet, LLC | Terminated |
| 03/16/2021 | Bravo Packing, Inc. | Pet food: Performance Dog, beef, green tripe, and bone | Due to Potential *Salmonella* and *Listeria monocytogenes* | Bravo Packing, Inc. |  |
| 04/10/2020 | Redbarn, Chewy Louie, Dentley's, and Good Lovin' | Bully Stick dog food | Potential to be contaminated with *Salmonella* | Redbarn Pet Products, LLC | Terminated |
| 02/15/2018 | ZooLogics and more | ZooLogics Duck with Vegetable Meals for Dogs, ZooLogics Chicken with Vegetable Meals for Dogs and more | Positive findings of Shiga toxin-producing E. coli O128, Salmonella and/or *Listeria monocytogenes* | Arrow Reliance Inc. dba Darwin’s Natural | Terminated |
| 03/11/2020 | Columbia River Natural Pet Foods | Frozen meat product for dogs and cats | Potential to be contaminated with *Listeria monocytogenes* | Columbia River Natural Pet Foods | Terminated |
| 03/11/2020 | Columbia River Natural Pet Foods | Dog and Cat fresh frozen meats | Due to Possible *Salmonella* and L. Mono Contamination | Columbia River Natural Pet Foods | Terminated |
| 02/07/2018 | Evanger’s | 12oz Hunk of Beef Dog food | Pentobarbital | Evanger’s Dog & Cat Food Co. |  |
| 09/24/2019 | TDBBS | Pig ear pet treat | *Salmonella* contamination | TDBBS LLC |  |
| 09/26/2019 | TDBBS | Pig ear pet treat | Possible *Salmonella* contamination | TDBBS LLC |  |
